# Increased expression of Ero1L-alpha in healing fetal wounds

**DOI:** 10.1186/1756-0500-4-175

**Published:** 2011-06-06

**Authors:** Phillip H Gallo, Latha Satish, Sandra Johnson, Sandeep Kathju

**Affiliations:** 1 Center for Genomic Sciences, Allegheny-Singer Research Institute, Allegheny General Hospital, Pittsburgh, PA 15212, USA

## Abstract

**Background:**

Adult mammalian tissues heal injury to the skin with formation of scar; this process quickly seals an injured area, however, excessive scar formation can become a source of persistent pathology, interfering with multiple vital functions. In contrast, mammalian fetal tissue can heal without scar formation. We previously sought to model scarless healing in a rabbit fetal skin wound and identified gene products differentially expressed during fetal wound healing through PCR suppression subtractive hybridization (PCR SSH). One of these transcripts, previously identified simply as clone 11, showed putative increased expression in wounded fetal skin. This study establishes its identity as Ero1L-alpha and confirms its elevated expression in healing fetal wounds.

**Findings:**

After obtaining further sequence by 5' rapid amplification of cloned ends (RACE) we find that clone 11 is Ero1L-alpha. We determined that clone 11, a differentially expressed transcript in fetal wound healing, comprises the 3' untranslated region (UTR) of an approximately 4 kb transcript in rabbit tissues that corresponds to Ero1L-alpha. We showed that Ero1L-alpha is expressed predominantly as two transcripts in rabbit skin, namely a 1.6 kb transcript and the 4.0 kb transcript recovered in our PCR SSH screen via its 3' UTR sequence. However, a third transcript of 2.9 kb was also detected in Northern blots and was subsequently cloned and confirmed by 3' RACE. Knockdown of the clone 11 sequence in rabbit adult fibroblasts via siRNA resulted in significantly decreased Ero1L-alpha message expression. Increased expression of clone 11 (Ero1L-alpha) in a variety of cell types during the wound healing response was also confirmed by *in situ *hybridization.

**Conclusions:**

Ero1L-alpha is one of the previously unknown clones identified in a PCR SSH screen for genes differentially expressed in fetal wounded tissue. *In situ *hybridization confirms that Ero1L-alpha shows increased expression in multiple cell types after wounding of the fetal integument.

## Introduction

Mammalian skin has multiple critical functions including providing homeostasis and serving as a first line of defence against infection. When injury to the skin occurs, a complex series of processes initiate its repair [1,2]. Adult (postnatal) mammals heal injury to the skin with attendant scar formation [3-5]; this process quickly seals an injured area, but excessive scar formation can become a source of persistent pathology and can interfere with numerous vital functions. In contrast, mammalian fetal skin can heal without scar formation. Much research has focused on identifying the mechanisms underlying scarless fetal wound repair; these experiments have primarily compared adult and fetal wound healing by examining various growth factors/cytokines, extracellular matrix proteins, and chaperonins [6-13]. To date, no specific critical pathways determinative for scarless repair have been established.

We have previously examined scarless wound healing in fetal skin by incisional wound modelling in rabbits [14]. Using PCR suppression subtraction hybridization (PCR SSH) we identified transcripts exhibiting differential expression during the fetal wound healing response. Because PCR SSH compares two conditions across the entire expressome and recovers only fragments of gene products, numerous genes of unknown identity and/or function were recovered. One of the unidentified gene products, designated clone 11, was upregulated in pooled samples of fetal wounded tissue samples 12 hours post-wounding when compared to fetal unwounded control skin tissue. Herein, we present evidence identifying this transcript as Ero1L-alpha and confirming its elevated expression with *in vivo *studies.

## Materials and methods

### Source Tissues and Fibroblast Culture

All animal protocols were reviewed and approved by the Institutional Animal Care and Use Committee (IACUC); details of animal sample collection were as described in *Kathju et al *[14]. ~1 cm incisional wounds were placed on the dorsums of fetal rabbits at 20-21 days gestation, then harvested 8 days later (as well as unwounded fetal control skin). For *in situ *hybridization studies, fetal wounded and unwounded control tissues were stored in 10% Neutral Buffered Formalin (up to 7 days) before embedding; processing, paraffin embedding, and sectioning of samples were performed by Research Histology Services (Pittsburgh, PA) using standard conditions.

For fibroblast culture, samples of unwounded control skin were obtained from adult and fetal rabbits and were minced into small pieces within 30 minutes after dissection. The tissue samples were washed extensively in PBS containing 1X antibiotic/antimycotic solution (containing penicillin, streptomycin, and amphotericin B; Invitrogen Corporation, Carlsbad, CA) and then placed in RPMI 1640 medium (Invitrogen) containing 10% fetal bovine serum (FBS, Gemini Bio-Products) and 1× antibiotic/antimycotic solution (Invitrogen). The cultures were left undisturbed for 7 days in a 37°C incubator containing 5% CO_2 _supplement. The fibroblast outgrowths observed after a week from primary cultures were sub-cultured immediately using 0.5% Trypsin EDTA (Invitrogen). Once the cells reached 90% confluence, they were either: 1) passaged once more before total RNA was isolated from passage three adult and fetal fibroblasts, or 2) plated onto 6-well plates, transfected with 100 μM siRNA (sequences in Table 1) and 4 μl Lipofectamine 2000 (Invitrogen) as previously described [15], and grown for 2 days before purification of total RNA.

**Table 1:** Primers, probes, siRNAs used

Primer Number/Name (from Figure 2A, if applicable)	Direction	Primer Sequence
1	F	TCAGCCAGTGTGGAAGGAGGGA
2	F	AGGAGACGCAGAAGGCTGTTC
3	F	TTGGGTTGTTTTGGTGGTAGAAAGGT
4	F	GCCGACAGTCAGCAAGTTTGCTTTATC
5	F	GCAGGGCTTGTGGATGTAATGTG
6	R	CTGATGTCATTCCAGAAAGGAC
7	R	TAACAGCACAGTCCCTCCTTCCACAC
8	R	TCAGAGCAGTACCCAAACCCTG
9	R	CGGAACAGCAATGGAGTTGGTAAG
clone11 siRNA sense		rGrUrGrCrUrArCrArCrArGrArCrCrUrGrUrCrUTT
clone11 siRNA antisense		rArGrArCrArGrGrUrCrUrGrUrGrUrArGrCrArCTT
scrambled siRNA sense		rUrGrCrGrArUrArCrGrArCrArUrCrCrUrCrGrUTT
scrambled siRNA antisense		rArGrUrArCrCrUrGrCrUrGrGrGrUrCrArGrArATT
Ero1LA real time for	F	ACCTGAAGAGGCCTTGTCCTTT
Ero1LA real time rev	R	TCCATCAGGAACTTCATCAGATTG
Ero1LA real time probe	P	TGGAATGACATCAGCCAGTGTGGAAG
clone11 real time for	F	CTCTGAAAACATGACTCCCTCCTT
clone11 real time rev	R	AGGAGTCTGGCTTTCTCCTGAA
clone11 real time probe	P	CACCGCTCTGTGACCTCCTGAAC
GAPDH real time for	F	CTCTGAAAACATGACTCCCTCCTT
GAPDH real time rev	R	CCTCGGTGTAGCCCAGGAT
GAPDH real time probe	P	AAGCAGGCATCCGAGGGCCC

### RNA extraction/purification of samples

For all tissue culture RNA purifications, total RNA was obtained using the RNeasy Micro Kit (Qiagen Inc. USA, Valencia, CA) following manufacturer's protocols with a DNase treatment step. The quality of total RNA extracted from fetal and adult tissue and fibroblasts was examined by capillary electrophoresis using an Agilent 2100 BioAnalyzer (Agilent Technologies Inc., Palo Alto, CA), and the quantity determined using the OD_260_/OD_280 _ratio measured using a ND-1000 spectrophotometer (Nanodrop Technologies Inc., Wilmington, DE).

### RACE

Rapid amplification of cloned ends (RACE) was used to obtain 3' UTR sequence for the various Ero1L-alpha clones. The GeneRacer kit with AMV reverse transcriptase (Invitrogen) was used for 3' RACE following manufacturer's directions; 2 μg of total RNA from fetal control skin was used as the source RNA. All subsequent PCR was done using the AccuPrime HF PCR system (Invitrogen) and using the primers listed in Table 1 following manufacturer's directions. Cloned amplimers from RACE reactions were then sequenced.

Primers for Ero1L-alpha were designed from the predicted rabbit sequence for Ero1L-alpha (ENSOCUG00000012632) from Ensembl [16] and primers for GAPDH were designed from the NCBI rabbit GAPDH sequence (NM_001082253) [17].

### DIG RNA probes

DNA constructs for DIG RNA probes were prepared by either DNA digests of plasmids bearing subcloned inserts or by direct PCR of cloned and sequenced probes; DNA templates were gel extracted before use. DIG RNA probes were prepared using the DIG RNA labelling kit (Roche, Indianapolis, IN) and T3 RNA Polymerase (Promega Corporation, Madison, WI). RNA was purified using the RNeasy Micro Kit (Qiagen) following manufacturer's protocols.

### Northern blot

Northern blots were either commercially prepared (Zyagen, San Diego, CA) or prepared using passage three fetal and adult fibroblast RNA. Northern blots were prepared using reagents from the NorthernMax kit (Applied Biosystems/Ambion, Austin, TX) and with the NorthernMax Loading Dye without ethidium bromide. Total RNA was used as the source for preparing mRNA using the Oligotex kit (Qiagen). 100 nanograms of mRNA and RNA marker (Invitrogen) were separated on 1% denaturing formaldehyde gels and transferred to a positively charged nylon membrane (Roche). RNA was cross-fixed to the membrane using a UV light box (SpectroLinker XL-1000, optimal cross-link settings).

Blots were rinsed with DEPC-treated water, and then prehybridized in DIG EasyHyb buffer (Roche) following manufacturer's protocol. Blots were hybridized overnight at 68°C in DIG EasyHyb with 100 ng DIG-labeled probe. Blots were washed for two 5 minute low stringency washes in 2 × SSC, 0.1% SDS at room temperature followed by two 20 minute high stringency washes in 0.2 × SSC, 0.1% SDS at 68°C. All blocking, washing and detection of DIG were done with the DIG wash and Block kit (Roche) and CDP-Star (Roche) following manufacturer's protocols.

### Quantitative real time RT-PCR

The primer sets for rabbit clone 11 and rabbit Ero1L-alpha were designed using the initial sequence for clone 11 obtained by PCR SSH, plus additional sequence obtained through 5' RACE and 3' RACE, as well as the predicted rabbit sequence for Ero1L-alpha (ENSOCUG00000012632) from Ensembl [16]. The Taqman primers/probes reported in Table 1 were designed using Primer Express software (Applied Biosystems, Foster City, CA). Initial RT-PCR assays on 100 ng of fetal unwounded control RNA were used to verify that each of the primer sets was detectable in fetal tissue and resulted in only a single amplicon of the expected molecular weight. Primer sequences for the rabbit GAPDH (used as an internal control) were previously published [14]. All primers and fluorocoupled Taqman probes were purchased from Integrated DNA Technologies (Coralville, IA).

100 ng of total RNA from samples was used for reverse transcriptase (RT) reaction (using gene-specific reverse primer and 10 μl of total volume); for subsequent real time PCR assays, 1.5 μL of RT reaction, 800 nM of each primer, and 160 nM of the appropriate probe (final concentrations) in a total volume of 15 μl were used. The remaining protocol parameters for RT reaction and real time PCR were followed as previously described [14]. Using the comparative critical cycle (Ct) method and using GAPDH as the endogenous control, the expression levels of the target genes were normalized and the relative abundance was calculated. Results shown are representative of three independent experiments performed in triplicate; statistical analysis for significance was performed using a Student's t-test.

### *in situ *Hybridizations

Sectioned slides of unwounded fetal skin or wounded fetal skin at 8 days post-injury were processed as described [18], except using paraffin embedded sections. Slides were deparaffinated using three xylene washes and passed through an ethanol series, with a final wash in 1× PBS. Slides were then processed as described [18], using a 48 hour hybridization at 58°C. Slides were counterstained with 0.1% Nuclear FastRed (Vector Labs, Burlingame, CA) and mounted with aqueous ImmuMount (ThermoFisher, Pittsburgh, PA) before photographing.

## Results/Discussion

**Figure 1:**
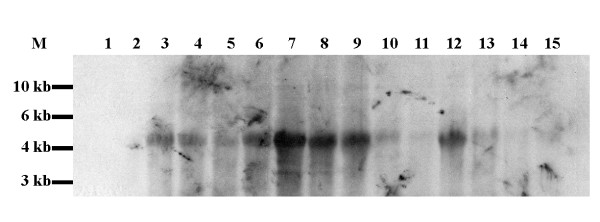
**Clone 11 expression reveals a single 4 kb transcript across most tissue types**. Northern blot analysis of clone 11 using adult total RNA, rabbit multi-tissue samples, and a non-radioactive DIG RNA probe; the lanes represent 1) brain, 2) stomach, 3) intestine, 4) colon, 5) liver, 6) lung, 7) kidney, 8) heart, 9) ovary, 10) skeletal muscle, 11) spleen, 12) testes, 13) thymus, 14) skin, and 15) pancreas. One predominant RNA transcript of approximately 4 kb was detected in most rabbit adult tissues (Figure 1), with highest expression in the lungs, kidneys, heart, ovaries, and testis and lowest expression in skin and pancreas.

Our original PCR SSH screen [14] recovered multiple fragments of gene sequence that are putatively over- or under-expressed in healing fetal wounds compared to unwounded fetal control tissues, but to which an exact identity could not be assigned. To better characterize these gene fragments, we interrogated a commercially available rabbit multi-tissue Northern blot with non-radioactive DIG probes directed against clones identified in our screen. When the Northern blot was hybridized with a probe directed against one gene product from our screen, previously identified simply as clone 11, a single major transcript of approximately 4 kb was detected in all rabbit adult tissues (Figure 1), with highest levels of expression in the lungs, kidneys, heart, ovaries, and testis, and lower levels of expression in the pancreas.

We originally obtained only several hundred base pairs of clone 11 sequence from our PCR SSH screen. We then undertook both 5' and 3' RACE to obtain further contiguous clone 11 sequence in the hopes of establishing its identity. Using the Ensembl rabbit database and BLAST, we determined that this expanded clone 11 sequence was derived from a rabbit genomic region approximately 2.5 kb from the predicted gene Ero1L-alpha. In humans, two transcripts of Ero1L-alpha are known to exist; both contain a 1290 bp coding region but differ in the length of their 3' UTRs, with the longer one approximating 5.3 kb in total length.

We therefore hypothesized that clone 11 in the rabbit was the extended 3' UTR form of Ero1L-alpha. Using the rabbit predicted Ero1L-apha sequence from Ensembl (ENSOCUG00000012632), we designed primers to amplify (by RT-PCR) a large portion of the Ero1L-alpha coding region from rabbit fetal control RNA. This amplimer was subcloned and sequenced and matched the predicted rabbit sequence from Ensembl, with 92% sequence similarity to human Ero1L-alpha and 97% predicted protein similarity within the coding region of Ero1L-alpha.

**Figure 2:**
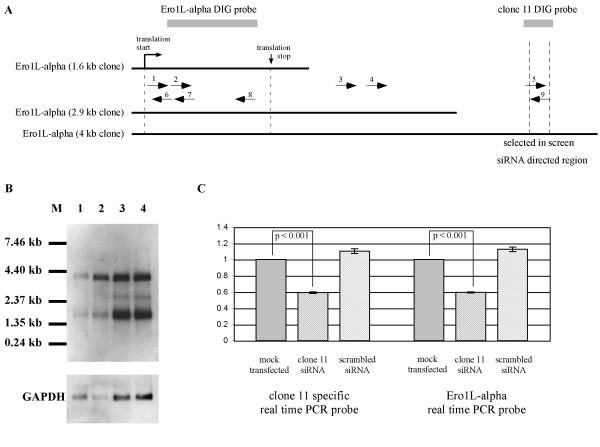
**Clone 11 represents sequence found in the 3' UTR of Ero1L-alpha**. (A) Schematic depiction of Ero1L-alpha message isoforms, with locations of probes and primers employed. (B) Northern blot analysis of Ero1L-alpha using total RNA from rabbit fetal and adult skin and fibroblasts, and a non-radioactive DIG RNA probe; the lanes represent 1) fetal skin, 2) adult skin, 3) rabbit fetal fibroblast, and 4) rabbit adult fibroblast. (C) Rabbit adult fibroblasts were treated with siRNA against clone 11, as well as scrambled control siRNA and mock transfection. Extracted total RNA was then used as a template for real time PCR to assay for expression of Ero1L-alpha coding sequence and clone 11 target sequence. Both Ero1L-alpha and clone 11 were significantly reduced in siRNA-treated cells but not in control cells, indicating that clone 11 sequence represents Ero1L-alpha.

To confirm that the clone 11 sequence obtained from our screen was the transcript of Ero1L-alpha containing an extended 3' UTR, we designed siRNA against the clone 11 sequence and transfected rabbit adult fibroblasts with clone 11-specific siRNA, scrambled control siRNA, or mock-transfected. After 48 hours, the RNA purified from the various transfected fibroblast populations was reverse transcribed using a gene specific primer located within clone 11 sequence, and subjected to real time PCR with probes specific either to clone 11 or to the Ero1L-alpha coding region (Figure 2C). Due to the use of clone 11 sequence as the primer for reverse transcription, located in the extended 3' UTR of the presumed Ero1L-alpha transcript isoform, the real time PCR probe designed against the coding sequence of Ero1L-alpha could only detect the extended 3' UTR transcript. We found that the siRNA against clone 11 significantly reduced detection of both clone 11-specific and Ero1L-alpha coding sequence-specific real time PCR probes. Neither scrambled siRNA nor mock transfection resulted in any Ero1L-alpha message decrease, confirming that our clone 11 sequence is the 3' terminus of the extended 4 kb Ero1L-alpha transcript. As a further confirmation of these results, mouse NIH 3T3 cells were transfected with siRNA against sequence corresponding to clone 11, and demonstrated a similar reduction in Ero1L-alpha coding transcript (data not shown).

Using a DIG-labelled probe derived from rabbit Ero1L-alpha coding sequence, we sought to determine which transcript isoforms are expressed in fetal and adult skin tissues and fibroblasts, and to confirm that Ero1L-alpha is expressed as two major transcripts in the rabbit as was previously reported in humans. Using a Northern blot prepared with total RNA isolated from fetal and adult unwounded skin tissues and from fetal and adult fibroblasts in culture, two transcripts of the expected molecular weights (~1400 bp and ~ 4 kb) were in fact observed in all samples (Figure 2B). Interestingly and surprisingly, a third minor message isoform corresponding to some 2.9 kb in length was also detected. We have subsequently used 3' RACE (using primers within the Ero1L-alpha coding sequence) to amplify and subclone the 3' end of this isoform, and confirm that it carries coding sequence identical to the other two transcripts, but with an intermediate length 3' UTR (data not shown). A full schematic, showing the various Ero1L-alpha message isoforms, together with locations of probes and primers is depicted in Figure 2A.

**Figure 3:**
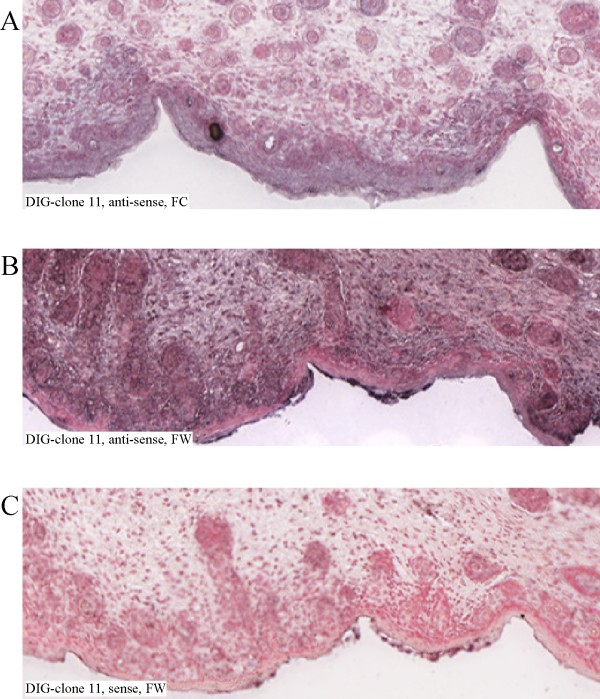
**Increased expression of Ero1L-alpha in healing fetal wound**. *In situ *hybridization using probe against clone 11 sequence reveals that Ero1L-alpha is over-expressed in multiple cell types after fetal skin wounding (3B) compared to unwounded control skin (3A). (3C): healing fetal wound tissue probed with a sense probe to clone 11, which would not be expected to hybridize to the target sequence. All images are at 5 × magnification.

Finally, we sought to determine if the long (4 kb) variant of Ero1L-alpha is indeed increased after fetal wounding, as would be expected from its survival in the PCR SSH screen, and in which cell types this might be evident. To address this question, fetal control and wounded tissues were paraffin embedded, sectioned onto slides, and used for *in situ *hybridization (Figure 3). DIG-labeled RNA probes against the original clone 11 sequence were used to determine that healing fetal wound tissue displayed a greater level of expression as compared to control tissue from unwounded littermates (Figure 3). A substantially higher intensity of staining was observed when antisense probe was used on wounded fetal tissue (Figure 3B) compared to unwounded fetal control tissue (Figure 3A) Minimal staining was observed in wounded tissue using sense probe (Figure 3C) attesting that the assay is detecting the correct RNA target. Interestingly, the 4 kb Ero1L-alpha isoform appears to show increased expression in almost all cell types within or adjacent to the zone of healing injury. To ensure that our observed over-expression of Ero1L-alpha was not due to differences in RNA levels due to sample preparation, a rabbit probe against GAPDH was generated and tested in *in situ *hybridization as well. This probe demonstrated no major difference in staining intensity between the fetal control and wounded samples (data not shown), confirming that the increased expression seen in Ero1L-alpha is a specific event.

Ero1L-alpha plays a major role in the oxidative protein folding pathway in the endoplasmic reticulum, enabling proteins to form disulfide bonds [19], and is specifically up-regulated in response to hypoxic conditions [20]. It has not been previously implicated in wound healing, but it is not surprising that its expression may be elevated in response to some hypoxic or ischemic stimulus at the zone of injury. It is interesting that we recovered only the 4 kb variant of Ero1L-alpha transcript in our PCR SSH screen, with its attendant lengthy 3' UTR. Such UTR sequences have been found in other systems to regulate gene expression through post-transcriptional mechanisms [21-24], and it may be that a similar phenomenon is occurring here. More study will be required to fully explicate the specific functional and regulatory significances of the multiple Ero1L-alpha isoforms we have identified.

## Conclusion

We conclude that one of the previously unidentified gene fragments recovered in a PCR SSH screen examining fetal wound healing, clone 11, is a 4 kb isoform of Ero1L-alpha, featuring an extended 3' UTR sequence. Ero1L-alpha is expressed as three distinct mRNA isoforms in fetal and adult skin tissues and fibroblasts, including a novel 2.9 kb variant. The 4 kb isoform of Ero1L-alpha shows increased expression in multiple cell types after fetal integumentary wounding by *in situ *hybridization.

## Competing interests

The authors declare that they have no competing interests.

## Authors' contributions

PHG, LS and SK designed the study. PHG performed the majority of the experimental work. SJ assisted with animal surgery, sample collection and RNA purification. LS derived fibroblast cultures and performed initial siRNA experiments. PHG, SK, and LS drafted the manuscript. All authors critically reviewed the final manuscript
